# Comparison of Acute Kidney Injury After Robot-Assisted Laparoscopic Radical Prostatectomy Versus Retropubic Radical Prostatectomy: A Propensity Score Matching Analysis

**DOI:** 10.1097/MD.0000000000002650

**Published:** 2016-02-08

**Authors:** Eun-Young Joo, Yeon-Jin Moon, Syn-Hae Yoon, Ji-Hyun Chin, Jai-Hyun Hwang, Young-Kug Kim

**Affiliations:** From the Department of Anesthesiology and Pain Medicine, Asan Medical Center, University of Ulsan College of Medicine, Seoul, Republic of Korea.

## Abstract

Acute kidney injury (AKI) is associated with extended hospital stay, a high risk of progressive chronic kidney diseases, and increased mortality. Patients undergoing radical prostatectomy are at increased risk of AKI because of intraoperative bleeding, obstructive uropathy, older age, and preexisting chronic kidney disease. In particular, robot-assisted laparoscopic radical prostatectomy (RALP), which is in increasing demand as an alternative surgical option for retropubic radical prostatectomy (RRP), is associated with postoperative renal dysfunction because pneumoperitoneum during RALP can decrease cardiac output and renal perfusion. The objective of this study was to compare the incidence of postoperative AKI between RRP and RALP.

We included 1340 patients who underwent RRP (n = 370) or RALP (n = 970) between 2013 and 2014. Demographics, cancer-related data, and perioperative laboratory data were evaluated. Postoperative AKI was determined according to the Kidney Disease: Improving Global Outcomes criteria. Operation and anesthesia time, estimated blood loss, amounts of administered fluids and transfused packed red blood cells, and the lengths of the postoperative intensive care unit and hospital stays were evaluated. Propensity score matching analysis was performed to reduce the influence of possible confounding variables and adjust for intergroup differences between the RRP and RALP groups.

After performing 1:1 propensity score matching, the RRP and RALP groups included 307 patients, respectively. The operation time and anesthesia time in RALP were significantly longer than in the RRP group (both *P* < 0.001). However, the estimated blood loss and amount of administered fluids in RALP were significantly lower than in RRP (both *P* < 0.001). Also, RALP demonstrated a significantly lower incidence of transfusion and smaller amount of transfused packed red blood cells than RRP (both *P* < 0.001). Importantly, the incidence of AKI in RALP was significantly lower than in RRP (5.5% vs 10.4%; *P* = 0.044). Furthermore, the length of hospital stay in RALP was also significantly shorter (*P* < 0.001).

The incidence of AKI after RALP is significantly lower than after RRP. RALP can therefore be a better surgical option than RRP in terms of decreasing the frequency of postoperative AKI.

## INTRODUCTION

Radical prostatectomy is a standard surgical treatment for clinically localized prostate cancer.^1^ Since retropubic radical prostatectomy (RRP) was developed in 1945,^2^ it has been optimized as the surgical technique of choice to reduce short-term and long-term complications and improve functional results in terms of both urinary continence and erectile function.^3–6^ Laparoscopic prostatectomy was developed and refined in 1999 with the intention of reducing the invasiveness of traditional open surgery and improving functional results, but the outcomes of laparoscopic prostatectomy patients were not much improved over RRP.^7–9^ The development of robot-assisted laparoscopic radical prostatectomy (RALP) soon followed laparoscopic prostatectomy in an attempt to reduce the difficulty involved in performing complex laparoscopic urologic procedures. RALP has been known to be related to lower blood loss and blood transfusion rates and shorter hospital stays in comparison with RRP.^10^ However, RALP requires a longer operation time and results in worse physiological changes due to pneumoperitoneum and the steep Trendelenburg position in comparison with RRP.^11,12^ The glomerular filtration rate, renal blood flow, and urine output can thereby decrease with intraperitoneal carbon dioxide insufflation during RALP.^13–15^

Acute kidney injury (AKI) is increasingly recognized as a serious postoperative complication and is linked to increased health costs and adverse outcomes including progression to chronic kidney disease and death.^16,17^ However, there have been no comparable studies to date on the evaluation of AKI between RRP and RALP. Therefore, we aimed in our current study to compare the incidence of postoperative AKI based on Kidney Disease: Improving Global Outcomes (KDIGO) criteria between RRP and RALP using propensity score matching analysis.

## METHODS

**FIGURE 1:**
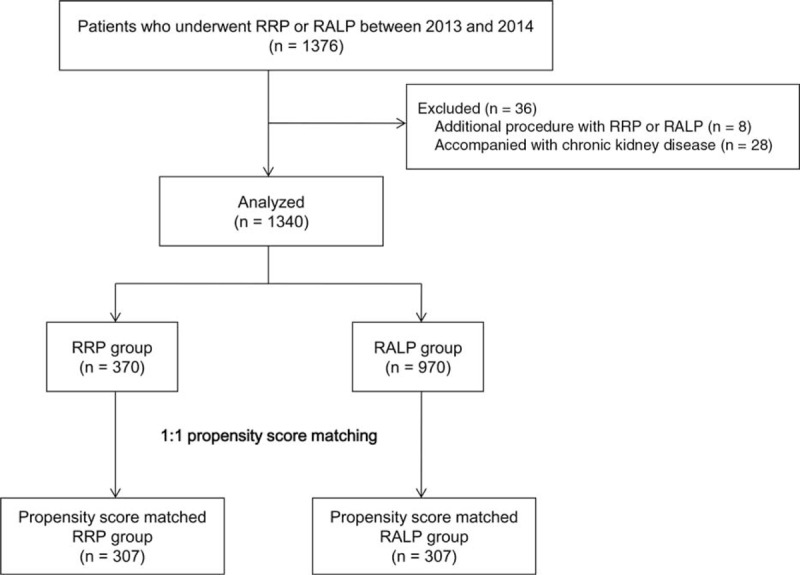
Study flow diagram. RALP = robot-assisted laparoscopic radical prostatectomy, RRP = retropubic radical prostatectomy.

Following approval by the institutional review board of Asan Medical Center, the records of all patients who underwent RRP or RALP at Asan Medical Center, Seoul, Republic of Korea between January 2013 and December 2014 were searched. Of the 1376 searched patients, we excluded those who underwent additional procedures (n = 8) or had any history of chronic kidney disease (n = 28). A final cohort of 1340 patients was included in the present study (Figure 1).

### Anesthetic Technique

Routine monitorings, including electrocardiography, noninvasive blood pressure monitoring, and pulse oximetry were performed before induction. General anesthesia was induced with propofol and rocuronium and maintained by sevoflurane-nitric oxide or sevoflurane-remifentanil. Following tracheal intubation, the invasive arterial blood pressure, body temperature, and hemoglobin concentration were additionally monitored. Fluids were administered using crystalloid (Hartmann's solution or Plasmalyte) and colloid (Volulyte). Systolic arterial blood pressure was maintained at 90 mm Hg or more during surgery. If systolic arterial blood pressure was less than 80 mm Hg, vasoactive drugs (ephedrine, phenylephrine, or norepinephrine) were administered. The hemoglobin concentration was maintained at 7 g/dL or more; if the hemoglobin concentration was less than 7 g/dL, a packed red blood cell transfusion was planned.

### Surgical Technique

The key procedures for RRP and RALP were performed according to the standard protocols of our institution.^18^ For RRP, a lower midline abdominal incision was made and the endopelvic fascia was opened from the base of the prostate to the apex. For RALP, pneumoperitoneum was established using a Veress needle, and 6 trocars were inserted. For RALP, the prostate was dissected using the antegrade approach. In both surgeries, bilateral pelvic lymph node dissection was performed, and the neurovascular bundles were spared for all potent patients. After the surgical specimen was removed, vesicourethral anastomosis was performed using a 20-Fr urethral catheter.

### Data Collection and Measurement

We collected information regarding the baseline characteristics and laboratory, intraoperative, and postoperative data from the computerized patient record system at our institution (Asan Medical Center Information System Electronic Medical Records). The baseline characteristics included age, height, weight, body mass index, comorbidities (eg, hypertension, diabetes mellitus, cardiac disease, and cerebrovascular disease), and the use of prescribed medications (beta-blockers and nonsteroidal anti-inflammatory drugs). Cardiac disease included ischemic heart disease and heart failure. Heart failure was defined as a history of any type of heart failure that was diagnosed by a cardiologist regardless of medication or decreased ejection fraction (ie, ejection fraction < 40%). Cerebrovascular disease was defined as a history of carotid artery stent or angioplasty, transient ischemic attack, stroke, or cerebral hemorrhagic event. Data on the status of patient's cancer including prostate-specific antigen (PSA) level and Gleason score were collected. The estimated glomerular filtration rate (eGFR), hematocrit, albumin, uric acid, and C-reactive protein levels were collected as preoperative laboratory data. eGFR was calculated using the 4-variable (age, sex, race, and serum creatinine) Modification of Diet in Renal Disease Study equation: eGFR = 186 × serum creatinine^−1.154^ × age^−0.203^ × [0.742 if female] × [1.210 if African-American].^19^

Intraoperative data included operation time, anesthesia time, estimated blood loss, volume of administered fluids, volume of transfused packed red blood cells, and the use of vasoactive drugs. The operation time was defined as the time between first incision and the end of the operation. Anesthesia time was defined as the time from anesthesia induction to tracheal extubation. Estimated blood loss was evaluated by the amount of lost red cell mass, which was calculated using the perioperative change in the hematocrit and transfused red cell mass using the following equation: lost red cell mass (mL) = patient's estimated blood volume (mL) × (preoperative hematocrit in % − postoperative hematocrit in %) + (transfused packed red blood cell in units × 250 (mL) × 0.6).^20^

### Primary and Secondary Endpoints

The primary endpoint of this study was the comparison of the incidences of AKI based on the KDIGO criteria between RRP and RALP. According to KDIGO criteria, AKI is defined as an increase in serum creatinine by 0.3 mg/dL or more within 48 hours or an increase in the serum creatinine by 1.5 times or more within the prior 7 days.^21^ However, in the present study, the urine output criterion was not included due to the inconsistency in urine output measurement. The secondary endpoints included the lengths of postoperative intensive care unit and hospital stay.

### Statistical Analysis

Before propensity score matching, we compared data between the RRP and RALP groups using the Chi-square test or Fisher exact test for categorical variables and the student *t* test or Mann–Whitney *U* test for continuous variables, as appropriate. Data are presented as the mean ± standard deviation, or number (percentage), as appropriate. We performed 1:1 propensity score matching analysis to reduce the influence of possible confounding variables and adjust intergroup differences between RRP and RALP groups. To determine the propensity score, a multiple logistic regression model was run using the following 17 variables: age, height, weight, body mass index, hypertension, diabetes mellitus, cardiac disease, cerebrovascular disease, taking beta-blockers or nonsteroidal anti-inflammatory drugs, PSA level, Gleason score, preoperative eGFR, hematocrit, albumin, uric acid, and C-reactive protein (Table 1). After performing 1:1 propensity score matching, continuous variables were compared using the paired *t* test or Wilcoxon signed-rank test, as appropriate, and categorical variables were compared using the McNemar test. Here, *P* < 0.05 was considered statistically significant. All statistical analyses were performed using SPSS for Windows (version 21; IBM Corp, Armonk, NY).

**TABLE 1:**
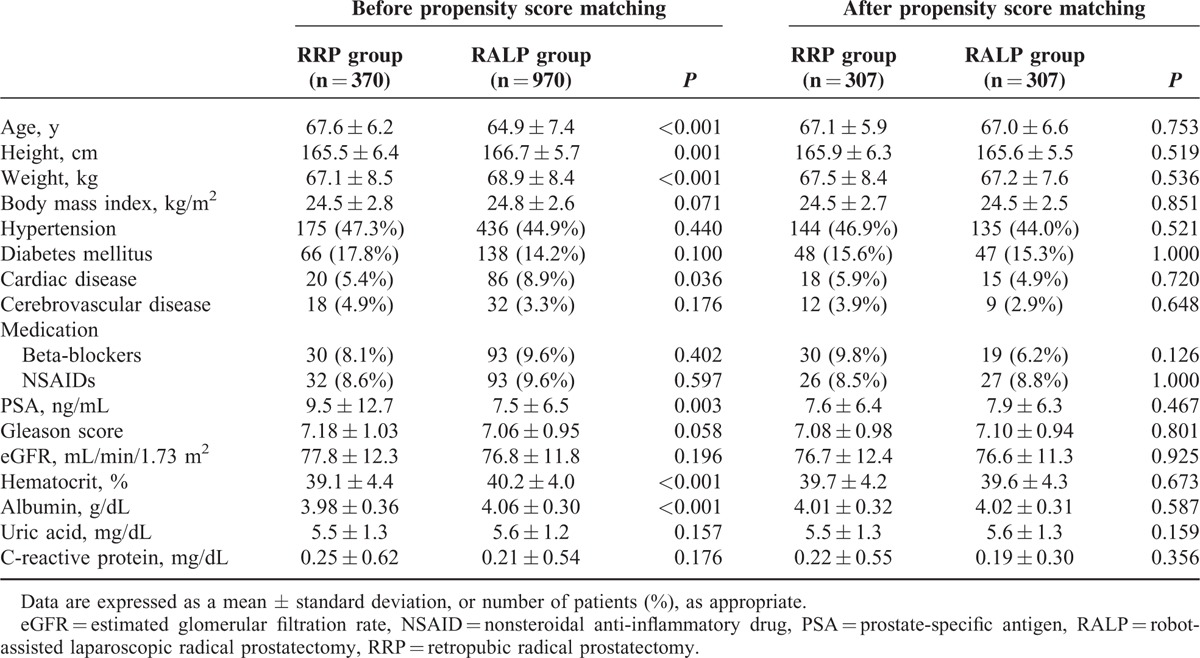
Demographic data, cancer-related data, and preoperative data between the RRP and RALP patients

## RESULTS

**FIGURE 2:**
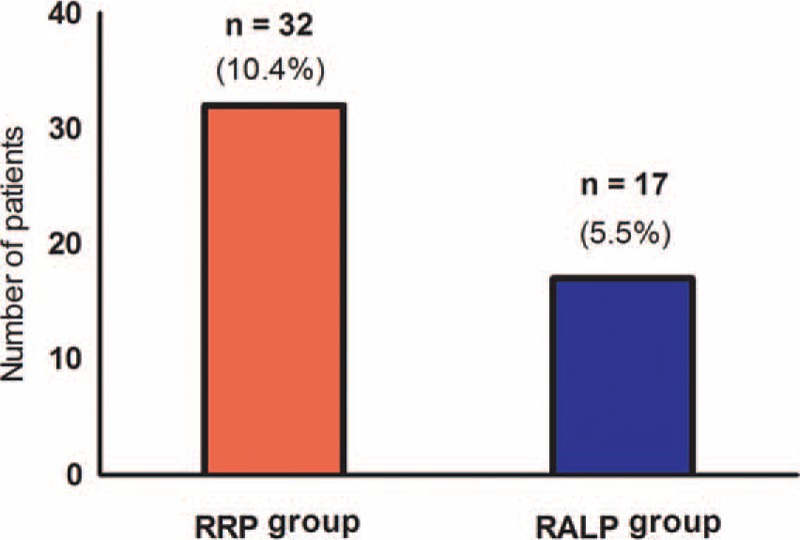
Incidences of postoperative AKI between the RRP and RALP groups. The incidence of AKI after RALP was significantly lower than after RRP. AKI =  acute kidney injury, RALP =  robot-assisted laparoscopic radical prostatectomy, RRP = retropubic radical prostatectomy.

A total of 1340 patients who underwent RRP (n = 370) or RALP (n = 970) were included in the current analyses. Age, height, weight, presence of cardiac disease, PSA, preoperative hematocrit, and albumin level demonstrated statistically significant differences between RRP and RALP groups (Table 1). After performing 1:1 propensity score matching analysis, there were no significant differences in demographic data, cancer-related data, or preoperative laboratory data between the RRP (n = 307) and RALP (n = 307) groups (Table 1). The operation time and anesthesia time in the RALP group were significantly longer than in the RRP group (both *P* < 0.001) (Table 2). However, the estimated blood loss and amount of administered fluids in the RALP group were significantly lower than in the RRP group (both *P* < 0.001) (Table 2). Also, the RALP group demonstrated a lower incidence of transfusion and smaller amount of transfused packed red blood cells than the RRP group (both *P* < 0.001) (Table 2). Importantly, the incidence of AKI in the RALP group was significantly lower than in the RRP group (5.5% [n = 17] vs 10.4% [n = 32]; *P* = 0.044) (Figure 2). Furthermore, the length of hospital stay in the RALP group was significantly shorter than in the RRP group (7.0 ± 2.5 days vs 8.8 ± 3.0 days; *P* < 0.001). However, there were no significant differences in the lengths of stay in the intensive care unit between the groups.

**TABLE 2:**
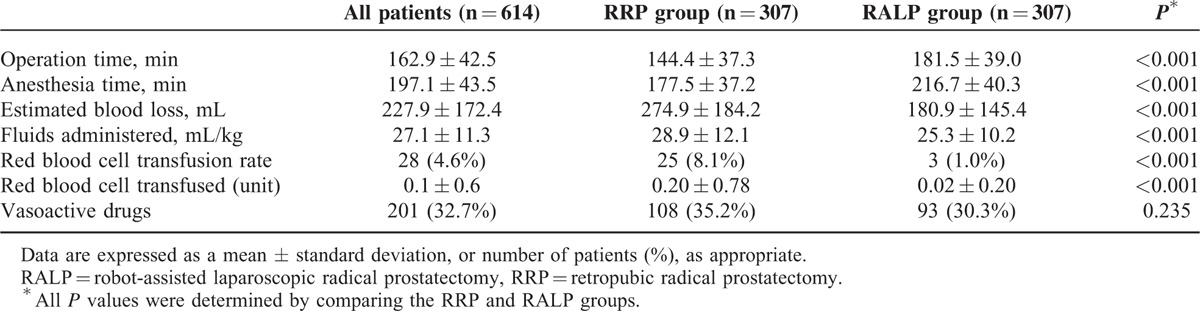
Intraoperative data for propensity score matched patients who underwent RRP or RALP

## DISCUSSION

In the present study, we found that the incidence of AKI after RALP was significantly lower than after RRP. The amounts of intraoperative blood loss and transfused packed red blood cells in RALP were also significantly lower, and the duration of hospital stay was significantly shorter in comparison with RRP.

Postoperative AKI is associated with increased costs, morbidity, and mortality and can increase the risk of progressive chronic kidney disease. Patients undergoing radical prostatectomy are at increased risk for AKI because of the common occurrences such as obstructive uropathy, older age, and preexistent chronic kidney disease, as well as intraoperative bleeding.^22^ Nevertheless, the exact incidence of AKI after radical prostatectomy using validated criteria have never been determined. Our present study provides the first information on the incidence of AKI after radical prostatectomy according to the KDIGO criteria, which can detect even acute subclinical increases in serum creatinine or decreases in eGFR after surgery.

The results of our current analyses showed a postoperative AKI incidence of 5.5% after RALP and 10.4% after RRP. RALP often requires pneumoperitoneum with an intra-abdominal pressure of more than 15 mm Hg for better visualization of the surgical field and continues for more than 3 hours. Direct compression of the intra-abdominal vessels and renal parenchyma by pneumoperitoneum can decrease cardiac output, renal blood flow, the glomerular filtration rate, and urine output.^13–15^ These physiologic changes consequently stimulate the renin-angiotensin system and further decreases renal blood flow.^23,24^ All of these can contribute to the impairment of renal function. However, previous studies on this issue have reported that postoperative renal function is unaltered after RALP, even under using a pressure of 20 mm Hg for pneumoperitoneum.^25,26^ However, these earlier reports analyzed the change in the creatinine clearance or the value of eGFR to measure the differences between preoperative and postoperative renal function, instead of using validated criteria. Also, those studies analyzed only patients that underwent RALP, so the outcomes were not comparable to patients who underwent RRP. In our present study, we used the KDIGO criteria to define AKI, and its incidence between RRP and RALP groups were compared using propensity score matching analysis to reduce the influence of confounding variables and adjust intergroup differences between groups. Therefore, we believe that our present results are highly reliable for the evaluation of AKI after radical prostatectomy.

There are several comparative studies between RRP and RALP in terms of surgical, oncological, and functional outcomes. In line with a previous report,^10^ RALP demonstrated lower blood loss and blood transfusion rate in comparison with RRP. The steep Trendelenburg position for RALP under pneumoperitoneum improves visualization, and thus bleeding from the dorsal vein complex during surgery can be more easily controlled.^25^ Furthermore, the tamponade effect by pneumoperitoneum also contributes to reduced blood loss.^27^ Generally, anemia and blood transfusion are well-known important risk factors of renal injury after cardiac surgery.^28–31^ The pathogenesis is unclear, but several mechanisms have been suggested: high vulnerability to hypoxic injury to the kidney and iron-mediated oxidative kidney injury.^32–35^ A previous study of 8799 patients who underwent lower-extremity revascularization to investigate the effects of blood transfusion demonstrated that intraoperative blood transfusion was associated with renal failure, as well as morbidity and mortality.^36^ Another study of 1034 cardiac surgery patients reported that the patients who received a nonleukoreduced red blood cell transfusion were at a higher risk of acute kidney injury and in-hospital mortality than the patients who received leukoreduced red blood cell transfusion.^37^ Another retrospective study of trauma patients demonstrated that the transfusion of red blood cell stored for more than 14 days was associated with increased renal dysfunction and mortality.^38^ In our current study, the higher incidence of AKI after RRP may result from the decreases in cardiac output and renal perfusion, diminished oxygen delivery, and increased oxidative stress to the kidney that are associated with a larger amount of blood loss during RRP. Furthermore, the higher incidence of red blood cell transfusion during RRP may also be responsible for the higher incidence of AKI.

Rhabdomyolysis and urinary tract obstruction can occur after robotic or nonrobotic radical prostatectomy and may be associated with development of AKI. Rhabdomyolysis can lead to glomerular filtration rate impairment. However, prostatectomy-related rhabdomyolysis is a very rare complication, and its incidence is reported to be 0.08%.^39^ In our current study, none of the patients with postoperative AKI previously had rhabdomyolysis or urinary tract obstruction after RRP or RALP.

The inevitable limitation of our current study comes from its retrospective design. Many confounders such as age, body mass index, comorbidities, and preoperative anemia may affect the accurate evaluation of the incidence of postoperative AKI. Also, previous studies show that cancer characteristics (eg, PSA level, Gleason score) might predict the risk of complications.^40,41^ Thus, we performed propensity score matching analysis for 17 confounding variables to minimize these biases. In addition, there are many difficulties in performing a randomized controlled trial to compare RALP and RRP because most patients are unwilling to accept the idea of randomization to a particular surgical treatment. Thus, propensity score matching analysis can be a reliable second-best strategy for comparing RALP and RRP.

In conclusion, postoperative AKI occurs at a lower incidence after RALP than RRP. This result provides valuable information on the additional benefit of RALP, which has many well-known advantages in comparison with RRP. Accordingly, RALP can be a better surgical option in terms of decreasing postoperative AKI than RRP.

## References

[1] Heidenreich A Bellmunt J Bolla M EAU guidelines on prostate cancer. Part 1: screening, diagnosis, and treatment of clinically localised disease. *Eur Urol* 2011; 59:61–71. 21056534 10.1016/j.eururo.2010.10.039

[2] Millin T Retropubic prostatectomy; a new extravesical technique; report of 20 cases. *Lancet* 1945; 2:693–696. 21007347 10.1016/s0140-6736(45)91030-0

[3] Walsh PC Anatomic radical prostatectomy: evolution of the surgical technique. *J Urol* 1998; 160:2418–2424. 9817395 10.1097/00005392-199812020-00010

[4] Steiner MS Continence-preserving anatomic radical retropubic prostatectomy. *Urology* 2000; 55:427–435. 10699626 10.1016/s0090-4295(99)00462-8

[5] Montorsi F Salonia A Suardi N Improving the preservation of the urethral sphincter and neurovascular bundles during open radical retropubic prostatectomy. *Eur Urol* 2005; 48:938–945. 16257111 10.1016/j.eururo.2005.09.004

[6] Graefen M Walz J Huland H Open retropubic nerve-sparing radical prostatectomy. *Eur Urol* 2006; 49:38–48. 16332409 10.1016/j.eururo.2005.10.008

[7] Guillonneau B Cathelineau X Barret E Laparoscopic radical prostatectomy: technical and early oncological assessment of 40 operations. *Eur Urol* 1999; 36:14–20. 10364650 10.1159/000019921

[8] Jacobsen NE Moore KN Estey E Open versus laparoscopic radical prostatectomy: a prospective comparison of postoperative urinary incontinence rates. *J Urol* 2007; 177:615–619. 17222646 10.1016/j.juro.2006.09.022

[9] Rassweiler J Schulze M Teber D Laparoscopic radical prostatectomy with the Heilbronn technique: oncological results in the first 500 patients. *J Urol* 2005; 173:761–764. 15711264 10.1097/01.ju.0000153486.94741.e5

[10] Novara G Ficarra V Rosen RC Systematic review and meta-analysis of perioperative outcomes and complications after robot-assisted radical prostatectomy. *Eur Urol* 2012; 62:431–452. 22749853 10.1016/j.eururo.2012.05.044

[11] Callery MP Soper NJ Physiology of the pneumoperitoneum. *Baillieres Clin Gastroenterol* 1993; 7:757–777. 8118072 10.1016/0950-3528(93)90014-j

[12] Gainsburg DM Anesthetic concerns for robotic-assisted laparoscopic radical prostatectomy. *Minerva Anestesiol* 2012; 78:596–604. 22415437

[13] Bishara B Karram T Khatib S Impact of pneumoperitoneum on renal perfusion and excretory function: beneficial effects of nitroglycerine. *Surg Endosc* 2009; 23:568–576. 18363060 10.1007/s00464-008-9881-4

[14] McDougall EM Monk TG Wolf JS Jr The effect of prolonged pneumoperitoneum on renal function in an animal model. *J Am Coll Surg* 1996; 182:317–328. 8605555

[15] Sassa N Hattori R Yamamoto T Direct visualization of renal hemodynamics affected by carbon dioxide-induced pneumoperitoneum. *Urology* 2009; 73:311–315. 19038429 10.1016/j.urology.2008.09.047

[16] Chertow GM Burdick E Honour M Acute kidney injury, mortality, length of stay, and costs in hospitalized patients. *J Am Soc Nephrol* 2005; 16:3365–3370. 16177006 10.1681/ASN.2004090740

[17] Hobson C Ozrazgat-Baslanti T Kuxhausen A Cost and mortality associated with postoperative acute kidney injury. *Ann Surg* 2015; 261:1207–1214. 24887982 10.1097/SLA.0000000000000732 PMC4247993

[18] Kim SC Song C Kim W Factors determining functional outcomes after radical prostatectomy: robot-assisted versus retropubic. *Eur Urol* 2011; 60:413–419. 21612859 10.1016/j.eururo.2011.05.011

[19] Levey AS Bosch JP Lewis JB A more accurate method to estimate glomerular filtration rate from serum creatinine: a new prediction equation. Modification of Diet in Renal Disease Study Group. *Ann Intern Med* 1999; 130:461–470. 10075613 10.7326/0003-4819-130-6-199903160-00002

[20] Lenoir B Merckx P Paugam-Burtz C Individual probability of allogeneic erythrocyte transfusion in elective spine surgery: the predictive model of transfusion in spine surgery. *Anesthesiology* 2009; 110:1050–1060. 19352170 10.1097/ALN.0b013e31819df9e0

[21] Kellum JA Lameire N Diagnosis, evaluation, and management of acute kidney injury: a KDIGO summary (Part 1). *Crit Care* 2013; 17:204. 23394211 10.1186/cc11454 PMC4057151

[22] Costalonga EC Costa ESVT Caires R Prostatic surgery associated acute kidney injury. *World J Nephrol* 2014; 3:198–209. 25374813 10.5527/wjn.v3.i4.198 PMC4220352

[23] Youssef MA saleh Al-Mulhim A Effects of different anesthetic techniques on antidiuretic hormone secretion during laparoscopic cholecystectomy. *Surg Endosc* 2007; 21:1543–1548. 17762955 10.1007/s00464-006-9166-8

[24] Myre K Rostrup M Eriksen M Increased spillover of norepinephrine to the portal vein during CO-pneumoperitoneum in pigs. *Acta Anaesthesiol Scand* 2004; 48:443–450. 15025606 10.1111/j.0001-5172.2004.00366.x

[25] Modi PK Kwon YS Patel N Safety of robot-assisted radical prostatectomy with Pneumoperitoneum of 20 mm Hg: a study of 751 patients. *J Endourol* 2015; 29:1148–1151. 25891967 10.1089/end.2015.0094

[26] Ahn JH Lim CH Chung HI Postoperative renal function in patients is unaltered after robotic-assisted radical prostatectomy. *Korean J Anesthesiol* 2011; 60:192–197. 21490821 10.4097/kjae.2011.60.3.192 PMC3071483

[27] De Carlo F Celestino F Verri C Retropubic, laparoscopic, and robot-assisted radical prostatectomy: surgical, oncological, and functional outcomes: a systematic review. *Urol Int* 2014; 93:373–383. 25277444 10.1159/000366008

[28] Karkouti K Wijeysundera DN Yau TM Acute kidney injury after cardiac surgery: focus on modifiable risk factors. *Circulation* 2009; 119:495–502. 19153273 10.1161/CIRCULATIONAHA.108.786913

[29] Karkouti K Beattie WS Wijeysundera DN Hemodilution during cardiopulmonary bypass is an independent risk factor for acute renal failure in adult cardiac surgery. *J Thorac Cardiovasc Surg* 2005; 129:391–400. 15678051 10.1016/j.jtcvs.2004.06.028

[30] Habib RH Zacharias A Schwann TA Role of hemodilutional anemia and transfusion during cardiopulmonary bypass in renal injury after coronary revascularization: implications on operative outcome. *Crit Care Med* 2005; 33:1749–1756. 16096452 10.1097/01.ccm.0000171531.06133.b0

[31] Ranucci M Romitti F Isgro G Oxygen delivery during cardiopulmonary bypass and acute renal failure after coronary operations. *Ann Thorac Surg* 2005; 80:2213–2220. 16305874 10.1016/j.athoracsur.2005.05.069

[32] Johannes T Mik EG Nohe B Acute decrease in renal microvascular PO2 during acute normovolemic hemodilution. *Am J Physiol Renal Physiol* 2007; 292:F796–F803. 17077389 10.1152/ajprenal.00206.2006

[33] Haase M Bellomo R Haase-Fielitz A Novel biomarkers, oxidative stress, and the role of labile iron toxicity in cardiopulmonary bypass-associated acute kidney injury. *J Am Coll Cardiol* 2010; 55:2024–2033. 20447525 10.1016/j.jacc.2009.12.046

[34] Hod EA Zhang N Sokol SA Transfusion of red blood cells after prolonged storage produces harmful effects that are mediated by iron and inflammation. *Blood* 2010; 115:4284–4292. 20299509 10.1182/blood-2009-10-245001 PMC2879099

[35] Ozment CP Turi JL Iron overload following red blood cell transfusion and its impact on disease severity. *Biochim Biophys Acta* 2009; 1790:694–701. 18992790 10.1016/j.bbagen.2008.09.010

[36] Tan TW Farber A Hamburg NM Blood transfusion for lower extremity bypass is associated with increased wound infection and graft thrombosis. *J Am Coll Surg* 2013; 216:1005–1014. 23535163 10.1016/j.jamcollsurg.2013.01.006 PMC5292257

[37] Romano G Mastroianni C Bancone C Leukoreduction program for red blood cell transfusions in coronary surgery: association with reduced acute kidney injury and in-hospital mortality. *J Thorac Cardiovasc Surg* 2010; 140:188–195. 20416894 10.1016/j.jtcvs.2010.03.022

[38] Weinberg JA McGwin G Jr Marques MB Transfusions in the less severely injured: does age of transfused blood affect outcomes? *J Trauma* 2008; 65:794–798. 18849793 10.1097/TA.0b013e318184aa11

[39] Wen T Deibert CM Siringo FS Positioning-related complications of minimally invasive radical prostatectomies. *J Endourol* 2014; 28:660–667. 24428586 10.1089/end.2013.0623

[40] Novara G Ficarra V D’Elia C Prospective evaluation with standardised criteria for postoperative complications after robotic-assisted laparoscopic radical prostatectomy. *Eur Urol* 2010; 57:363–370. 19944519 10.1016/j.eururo.2009.11.032

[41] Agarwal PK Sammon J Bhandari A Safety profile of robot-assisted radical prostatectomy: a standardized report of complications in 3317 patients. *Eur Urol* 2011; 59:684–698. 21324583 10.1016/j.eururo.2011.01.045

